# Natural Genetic Variation Screen in *Drosophila* Identifies Wnt Signaling, Mitochondrial Metabolism, and Redox Homeostasis Genes as Modifiers of Apoptosis

**DOI:** 10.1534/g3.119.400722

**Published:** 2019-09-30

**Authors:** Rebecca A. S. Palu, Elaine Ong, Kaitlyn Stevens, Shani Chung, Katie G. Owings, Alan G. Goodman, Clement Y. Chow

**Affiliations:** * Department of Human Genetics, University of Utah School of Medicine, Salt Lake City, UT 84112,; † School of Molecular Biosciences, and; ‡ Paul G. Allen School for Global Animal Health, Washington State University College of Veterinary Medicine, Pullman, WA 99164

**Keywords:** apoptosis, *Drosophila*, genetic variation, modifier genes

## Abstract

Apoptosis is the primary cause of degeneration in a number of neuronal, muscular, and metabolic disorders. These diseases are subject to a great deal of phenotypic heterogeneity in patient populations, primarily due to differences in genetic variation between individuals. This creates a barrier to effective diagnosis and treatment. Understanding how genetic variation influences apoptosis could lead to the development of new therapeutics and better personalized treatment approaches. In this study, we examine the impact of the natural genetic variation in the *Drosophila* Genetic Reference Panel (DGRP) on two models of apoptosis-induced retinal degeneration: overexpression of *p53* or *reaper* (*rpr*). We identify a number of known apoptotic, neural, and developmental genes as candidate modifiers of degeneration. We also use Gene Set Enrichment Analysis (GSEA) to identify pathways that harbor genetic variation that impact these apoptosis models, including Wnt signaling, mitochondrial metabolism, and redox homeostasis. Finally, we demonstrate that many of these candidates have a functional effect on apoptosis and degeneration. These studies provide a number of avenues for modifying genes and pathways of apoptosis-related disease.

Phenotypic heterogeneity is the driving force behind the Precision Medicine Initiative (Scriver and Waters 1999; Nadeau 2001; Queitsch *et al.* 2012; Gallati 2014). Patients suffering from the same genetic disorders can carry identical causal mutations but often display wildly variable phenotypes and symptom severity. A large part of this variation is due to inter-individual differences in genetic background, including silent cryptic genetic variation that is revealed upon disease or stress (Queitsch *et al.* 2012; Chow 2016). Understanding the role of this variation and the genes or pathways which modify disease will lead to improved personalized therapeutic predictions, strategies, and diagnostics.

One process implicated in many genetic disorders is programmed cell death or apoptosis (Elmore 2007; Sano and Reed 2013; Kurtishi *et al.* 2018). During normal development and tissue turnover, cells can receive both internal and external signals that trigger a programmed response which eventually results in the death of the cell (Elmore 2007). Because cell death is essential to cellular, tissue, and organismal homeostasis, disruption of apoptosis pathways can be catastrophic. Inhibition of apoptosis is an important step in transformation and cancer, while excess apoptosis, often activated by chronic cellular stress, is a primary cause of degeneration in different neuronal, retinal, muscular-skeletal, and metabolic diseases (Mattson 2000; Elmore 2007; Ouyang *et al.* 2012). As a result, an important area of therapeutic development is focused on targeting apoptosis without disrupting normal tissue homeostasis (Elmore 2007). Our previous work demonstrated that hereditary variation in apoptotic genes is associated with phenotypic variation in a model of retinal degeneration, suggesting that modifiers of apoptosis could serve as drug targets in degenerative diseases (Chow *et al.* 2016).

Model organism tools, such as the *Drosophila* Genetic Reference Panel (DGRP), enable the study of the impact of natural genetic variation on diseases and related pathways. The DGRP is a collection of ∼200 isogenic strains derived from a wild population, such that each strain represents one wild-derived genome (Mackay *et al.* 2012). The variation in the DGRP is well tolerated under healthy, non-disease conditions and allows for the identification of genetic polymorphisms that are associated with phenotypic variation in models of human disease (Chow and Reiter 2017). Importantly, the availability of full-genome sequence for these strains allows for genome-wide association analyses that link quantitative phenotypes with genetic variation and modifier genes.

In this study, we report the results of natural variation screens of *reaper*- (*rpr*) and *p53*-induced apoptosis (Figure 1). Overexpression of either of these genes leads to massive apoptotic activation (Hay *et al.* 1995; Jin *et al.* 2000). While there is a great deal of overlap between these pathways, they can each activate apoptosis independently. p53 is stabilized in response to DNA damage and initiates apoptosis by transcriptionally activating the inhibitor of apoptosis (IAP) inhibitors *rpr*, *grim*, and *hid* (Mollereau and Ma 2014) (Figure 1). P53 can also increase apoptosis by activating JNK signaling and stabilizing the IAP inhibitor Hid (Shklover *et al.* 2015). *rpr* is activated transcriptionally by either p53 or the JNK signaling cascade, which is induced downstream of oxidative, ER, and other cellular stresses (Kanda and Miura 2004; Shlevkov and Morata 2012) (Figure 1). We designed this study to identify genetic modifiers of general apoptosis induced by any cellular pathway, including modifiers that are specific to stress-induced, p53-independent pathways or specific to canonical p53-dependent pathways.

**Figure 1:**
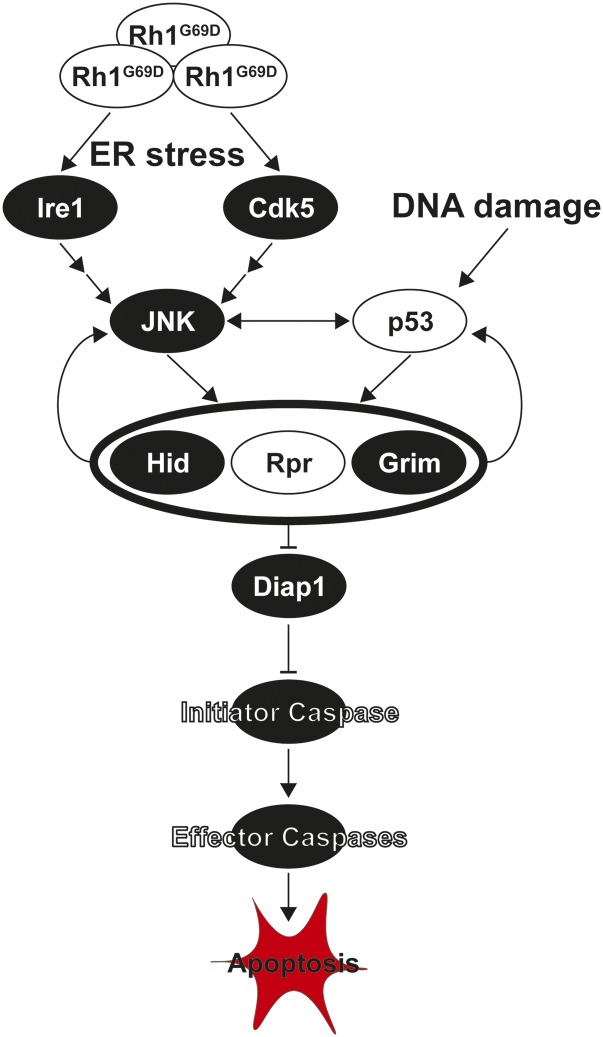
Activation of apoptosis through *p53* and *rpr*-associated pathways. Apoptosis is primarily initiated through either p53 or Jun-induced (JNK) transcriptional activation of the Inhibitor of Apoptosis (IAP, in *Drosophila* Diap) inhibitors *hid*, *rpr* and *grim*. While p53 is primarily activated by DNA damage and disruption of the cell cycle, JNK signaling is activated downstream of cellular stress, such as endoplasmic reticulum (ER) stress, through Ire1 and Cdk5. ER stress occurs when misfolding proteins, like the rhodopsin mutant *Rh1^G69D^*, accumulate in the ER (Chow *et al.* 2016). Expression of *rpr*, *grim*, and *hid* leads to inhibition of Diap1, releasing the inhibition on initiator caspases and allows for the activation of effector caspases and downstream apoptosis. Models used in this or previous studies of retinal degeneration in the DGRP are indicated in white.

We observed substantial phenotypic variation across the DGRP for both *rpr*- and *p53*-induced apoptosis. Using genome-wide association analysis, we identified a number of modifying pathways and genes, several of which have known roles in cell death pathways, neuronal development, neuromuscular diseases, and cancer. Using systems biology approaches, we also identified Wnt signaling, mitochondrial redox homeostasis, and protein ubiquitination/degradation as possible modifiers of apoptosis. Finally, we confirmed that reduction in the expression of many of these candidate modifier genes significantly alters degeneration. Our findings highlight several exciting new areas of study for apoptotic modifiers, as well as a role for stress-induced cell death in the regulation of degenerative disorders.

## Methods

### Fly stocks and maintenance

Flies were raised at room temperature (∼20-22°) on a diet based on the Bloomington Stock Center standard medium with malt. This diet consists of cornmeal, yeast, malt, and corn syrup and followed the recipe and proportions of the Bloomington cornmeal diet without the addition of soy flour. In this study, p53 is driven by the GAL4/UAS system and the strain contains a *GMR-GAL4* transgene and a *UAS-p53* transgene (*GMR > **p53*). *rpr* expression is driven directly by the *GMR* promoter sequences from a single transgene (*GMR-rpr)*. The strains containing *GMR-GAL4* and *UAS-p53* or *GMR-rpr* on the second chromosome have been previously described (Hay *et al.* 1995; Jin *et al.* 2000). In both cases, the apoptotic gene (*p53* or *rpr*) is overexpressed specifically in the developing *Drosophila* eye under the control of the *GMR* promotor. This leads to excess apoptosis in the eye imaginal disc and ultimately a small, degenerate eye once the adult ecloses. These are referred to as the apoptotic models throughout the manuscript. 204 strains from the DGRP were used for the *GMR > **p53* study (Table S1) and 202 were used for the *GMR-rpr* study (Table S2). In both cases virgin females carrying one of the apoptosis models were crossed to males of the DGRP strains. F1 progeny carrying *GMR > **p53* or *GMR-rpr* were collected and scored for eye size. As excess apoptosis leads to the degenerate eye phenotype, eye size was used as a proxy for cell death and variation in eye size as a proxy for variation in the degree to which apoptosis was activated during development. The following RNAi and control strains are from the Bloomington Stock Center: *swim* RNAi (55961), *CG3032* RNAi (57560), *LysRS* RNAi (32967), *αMan1a* RNAi (64944), *LIMK1* RNAi (62153), *hay* RNAi (53345), *CG1907* RNAi (38998), *Sema1a* RNAi (34320), *MED16* RNAi (34012), *bru1* RNAi (44483), *CycE* RNAi (33645), *shab* RNAi (55682), *CG31559* RNAi (64671), *Cyt-c-P* RNAi (64898), *Ir40A* (57566), *sif* RNAi (61934), control *attP40* (36304), and control *attP2* (36303). To test RNAi in the *GMR-rpr* model, a *GMR-GAL4* transgene was introduced into the strain through genetic crosses. While this effectively changes the genetic background of the GMR-rpr model, we control for this as much as possible by crossing this line to the *attP40* and *attP2* lines to generate the no RNAi controls.

### Eye size imaging

For eye images, adult females of the necessary genotypes were sorted by phenotype and sex under CO_2_ anesthesia. As cell death in these models occurs during development, eye size is stable through adulthood. Flies were aged at least 2 days to ensure they are fully mature, but not more than 7 days to prevent accumulated lethality due to a leaky GAL4 driver or UAS promotor, then flash frozen on dry ice. Left eyes were imaged for all measurements to maintain consistency. 10-15 eyes per strain were imaged using a Leica EC3 camera (Leica Microsystems) mounted on a FlyStuff Trinocular Microscope (Genesee Scientific) at 3X magnification. Camera settings were as follows: brightness-70%, gamma-0.70, saturation-140.00, EC3-14140014, configuration-last used, capture format-2048x1536, live format-1024x768, shading-none, sharpening-low. Eye area was measured in ImageJ as previously described (Chow *et al.* 2016). Two-dimensional eye area was measured as a proxy for eye size for each individual of each line. The outlines of the eyes were carefully traced using the freehand selection tool on ImageJ. We then used ImageJ to calculate the area in pixels enclosed by this selection and recorded that measurement as eye size in that individual. Average eye size for each line or transgenic strain was determined from 10-15 individual measurements.

### Phenotypic analysis and genome-wide association

For each *trans*-heterozygous DGRP*/GMR > **p53* or *GMR-rpr* line, eyes from 10-15 individual females were imaged and measured. For each model, a one-way ANOVA (R software) was used to determine if there was an effect of genetic background/strain on eye size. A genome-wide association (GWA) analyses was performed to identify genomic variants that are significantly associated with differences in eye size. Mean eye area was used for the GWA. GWA was performed as previously described (Chow *et al.* 2016). DGRP genotypes were downloaded from the website, http://dgrp.gnets.ncsu.edu/. Variants were filtered for minor allele frequency (≥ 0.05), and non-biallelic sites were removed. A total of 1,967,719 variants for *p53* and 1,962,205 variants for *rpr* were included in the analysis. Mean eye size for 204 F1 DGRP/*GMR > **p53* strains (representing 2953 flies) or 202 F1 DGRP/*GMR-rpr* strains (representing 2987 flies) were regressed on each SNP. To account for cryptic relatedness (He *et al.* 2014; Huang *et al.* 2014), GEMMA (v. 0.94) (Zhou and Stephens 2012) was used to both estimate a centered genetic relatedness matrix and perform association tests using the following linear mixed model (LMM):

y=α+xβ+u+ϵ

$$\text{u}∼\text{MVN}\_\text{n}\left(0,{\lambda \text{T}}^{\wedge }\left(-1\right)\text{K}\right)$$

$$\epsilon ∼\text{MVN}\_\text{n}\left({\text{0,T}}^{\wedge }\left(-1\right)\text{I\_n}\right)$$

where, as described and adapted from Zhou and Stephens 2012, y is the n-vector of mean eye sizes for the n lines, α is the intercept, x is the n-vector of marker genotypes, β is the effect size of the marker. u is a n x n matrix of random effects with a multivariate normal distribution (MVN_n) that depends on λ, the ratio between the two variance components, τ^(-1), the variance of residuals errors, and where the covariance matrix is informed by K, the calculated n x n marker-based relatedness matrix. K accounts for all pairwise non-random sharing of genetic material among lines. ϵ, is a n-vector of residual errors, with a multivariate normal distribution that depends on τ^(-1) and I_n, the identity matrix. Quantile-quantile (qq) plots demonstrate an appropriate fit to the LMM (Figure S1). Genes were identified from SNP coordinates using the BDGP R54/dm3 genome build. A SNP was assigned to a gene if it was +/− 1 kb from a gene body.

### Correlation Analysis

A Pearson Correlation test was performed to compare mean eye size between F1 DGRP/*GMR > **p53* strains and F1 DGRP/*GMR-rpr* strains of the same DGRP backgrounds (*i.e.*, the F1 of the RAL 227 crossed to *GMR > **p53* compared to the F1 of the RAL 227 crossed to *GMR-rpr*). The same test was used to compare the F1 DGRP/*GMR-rpr* and F1 DGRP/*GMR > Rh1^G69D^* (Chow *et al.* 2016), as well as F1 DGRP/*GMR > **p53* and F1 DGRP/*GMR > Rh1^G69D^*. Statistics were calculated using using R software.

### RNAi Validation

Virgin females of either the *GMR-GAL4*; *GMR-rpr* or *GMR > **p53* genotype were crossed to males carrying *UAS-RNAi* constructs targeting candidate modifier genes. Eye size of F1 progeny expressing both the apoptotic model and the RNAi construct was measured as described above. The eyes of 10-15 females were imaged and measured. Eye size from RNAi-carrying strains were compared directly to genetically matched *attP40* or *attP2* controls using a Dunnett’s multiple comparisons test.

### Bioinformatics Analysis

Genetic polymorphisms within an annotated gene were associated with that particular gene. Polymorphisms located outside of an annotated gene were associated with the closest annotated gene within 1 Kb. Polymorphisms located more than 1 Kb away from an annotated gene were labeled “intergenic” and not further considered. Information about candidate genes and their human orthologs was gathered from a number of databases including Flymine, Flybase, OMIM, and NCBI. Genetic interaction maps were generated using the GeneMANIA plugin on Cytoscape (version 3.6.1) (Shannon *et al.* 2003; Montojo *et al.* 2010). Gene Set Enrichment Analysis (GSEA) was run on all polymorphisms within 1 kb of a gene to generate a rank-list of genes based on their enrichment for significantly associated polymorphisms (see figshare for code). For GSEA analysis, polymorphisms within 1 kb of more than 1 gene were assigned to one gene based on an *a priori* list of exon, UTR, intron, and upstream or downstream. Genes were then assigned to GO categories. Using the entire ranked gene list instead of limiting the list by corrected p-value cut-off (Dyer *et al.* 2008), GSEA determines whether members of a given set of genes belonging to a specific GO category are randomly distributed throughout the ranked list, are found primarily at the top of the ranked list, or are found primarily at the bottom of the ranked list. GO categories enriched at the top of the list functionally describe the phenotype of the gene set. Calculation of enrichment score was performed as described (Subramanian *et al.* 2005). Categories with ES scores > 0 (enriched for associated genes with low p-values, ES scores ≤ 0 are unenriched), gene number > 3 (only see pathways represented by multiple variants), and corrected p-values <0.05 (significant associations) were included in the final output.

### Reagent and Data Availability

Strains and stocks are available upon request. Genomic sequence for the DGRP is available at http://dgrp.gnets.ncsu.edu/. Code and related guides for the GSEA analysis and supplemental material available at figshare: https://doi.org/10.25387/g3.9808379.

## Results and Discussion

### rpr- and p53-induced apoptosis is dependent on genetic background

**Figure 2:**
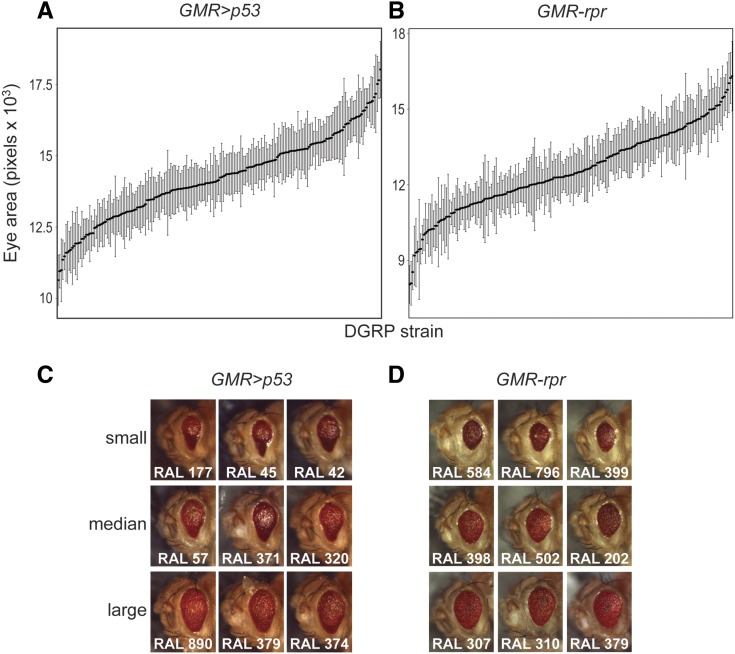
Apoptosis levels vary across genetic background in *p53* and *rpr* models of apoptosis-induced degeneration. Apoptosis induced by overexpression of *p53* (A) or *rpr* (B), as measured by adult eye size, varies across different genetic backgrounds. DGRP strains are arranged along the X-axis from smallest to largest. Each point represents the median eye size of an individual DGRP strain, while the error bars represent the standard deviation of eye size for that strain. Representative images of *GMR > **p53* eyes (C) or *GMR-rpr* eyes (D) in different DGRP backgrounds demonstrate the phenotypic variation quantified in panels A and B.

We used the *Drosophila* eye to model apoptosis. Expression of either *p53* or *rpr* in the ommatidial array of the developing eye imaginal disc results in massive cell death and smaller, rough adult eyes (Hay *et al.* 1995; Jin *et al.* 2000). The *rpr* model is induced by direct drive of the *GMR* promoter (*GMR-rpr*) on a second chromosome balancer. The *p53* model is induced using the *GAL4/UAS* system, where *GMR-GAL4* drives expression of *UAS-p53* (*GMR > **p53*). Importantly, in both of these models, adult eye size is an easily scorable, quantitative proxy for levels of apoptosis. The lines described serve as the donor strains (*GMR > p53/CyO* or *GMR-rpr,CyO/sna^sco^*) that we crossed to each DGRP strain. Females from the donor strains were crossed with males of each of 204 or 202 DGRP strains to generate F1 progeny that overexpressed *p53* or *rpr*, respectively, in the eye disc. The progeny received 50% of their genome from the maternal donor strain and 50% from the paternal DGRP strain. Therefore, we are measuring the dominant effect of the DGRP background on the *p53* or *rpr* retinal phenotype. This cross design is similar to a study of ER stress-induced degeneration (Chow *et al.* 2016) and a model of Parkinson’s Disease (Lavoy *et al.* 2018) we previously reported. The *GMR* promoter has been used in several previous publications (Chow *et al.* 2016; He *et al.* 2014) to drive overexpression of a *UAS*-transgene in the developing Drosophila eye imaginal disc. In all of these cases, the top candidates have been substantially different and highly dependent on the *UAS*-transgene. This suggests that the protein being overexpressed is more influential on the phenotype than the *GMR-GAL4* transgene. We examined eye size in the F1 progeny to determine the average eye size in individual genetic backgrounds (Figure 2A-D). As previous work has demonstrated a lack of correlation between variation in body size and variation in eye size in a model of developmental eye degeneration, there was no need to correct for overall body size (Chow *et al.* 2016).

We first tested the effect of sex on apoptosis in a pilot study. We measured eye area in at least ten females and ten males from eight different DGRP strains crossed to either the *p53* or *rpr* model. Eye size is positively correlated between males and females (Figure S2). Because variation is greater in females (Figure S2), we elected to focus on female eye size for the remainder of our analysis.

We found a significant effect of genetic background on eye size in the *GMR > **p53* model (*P* < 2.2 × 10^−16^) (Figure 2A,C, Table S1). Average eye size measured in pixels on ImageJ ranged from 10542 pixels (RAL812) to 17835 pixels (RAL374) (Table S1). Similarly, we found a significant effect of genetic background on eye size in the *GMR-rpr* model (*P* < 2.2 × 10^−16^), with median eye size ranging from 7957 pixels (RAL83) to 16884 pixels (RAL304) (Figure 2B,D, Table S2). For both the *GMR > **p53* and the *GMR-rpr* models, the variation in eye size within individual DGRP strains is substantially smaller than the variation observed between DGRP strains (Figure 2A-B, Table S1-S2).

We noted that the range in average eye size for the *GMR-rpr* model (8927 pixels) is greater than that seen in the *GMR > **p53* model (7293 pixels). This could be due to the greater involvement of *rpr* in a variety of stress-induced, *p53*-independent apoptotic pathways (Shlevkov and Morata 2012). Alternatively, it is possible that variation in *p53*-associated pathways is simply less well-tolerated than in *rpr*-associated pathways. It is also possible that the DGRP simply carries more variation affecting the *GMR-rpr* model than *GMR > **p53* model. Another possibility is that the differences in genetic background between these two models is driving differences in the way they respond to modifier variation in the DGRP. Finally, it is possible that the difference in range we observe are not statistically significant and is simply a stochastic artifact.

We observed qualitative differences between the apoptotic models, with flies expressing the *GMR > **p53* model displaying a teardrop-shaped eye (Figure 2C) and flies expressing the *GMR-rpr* model displaying a rounder eye (Figure 2D). These qualitative shapes were not subject to effects of genetic variation. The differences in eye shape noted between *GMR > **p53* and *GMR-rpr*, however, could be indicative of differences in the mechanisms by which apoptosis and degeneration progress in these two models. Alternatively, this could be evidence of the technical differences in the two models, since *p53* is driven by the *GAL4/UAS* system and *rpr* is driven directly by the *GMR* promotor. We saw no accumulation of necrotic tissue in strains experiencing severe degeneration, nor did we note obvious differences in pigmentation (Figure 2C,D). Eyes from all strains maintained the rough-eye phenotype that is characteristic of *p53* or *rpr*-induced degeneration, indicating that while modifying variation may reduce the amount of cell death in the eye imaginal disc, it cannot fully rescue the degenerative phenotype.

### Eye size in rpr-expressing DGRP lines correlates with eye size in other models of degeneration

**Figure 3:**
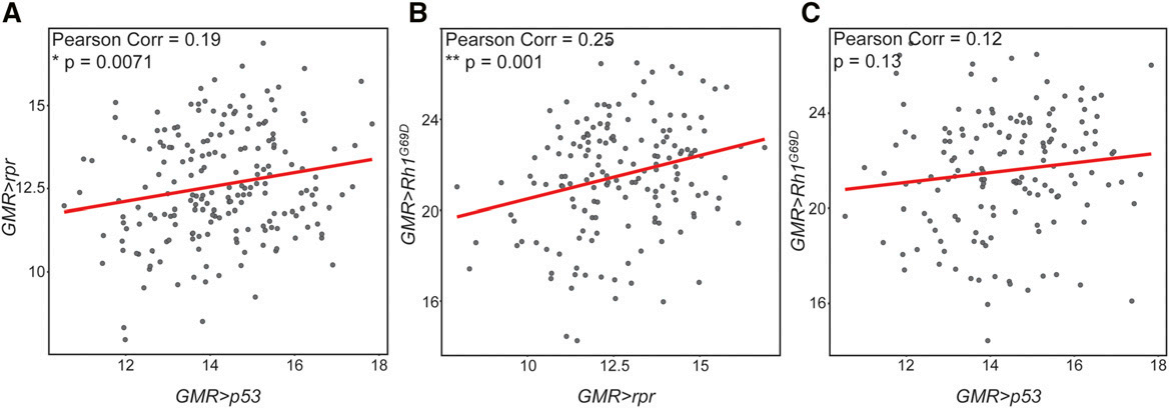
Eye size is correlated between *GMR-rpr* and both *GMR > **p53* and *GMR > Rh1^G69D^* models of degeneration. Correlation in mean eye size between the *GMR-rpr*, *GMR > **p53*, and *GMR > Rh1^G69D^* models across the DGRP. A. Eye size is significantly correlated in the same DGRP strains expressing *GMR-rpr* and *GMR > **p53* (r = 0.19, *P* = 0.0071). B. Eye size is significantly correlated in the same DGRP strains expressing *GMR-rpr* and *GMR > Rh1^G69D^* (r = 0.25, *P* = 0.001). C. Eye size is not correlated in the same DGRP strains expressing *GMR > **p53* and *GMR > Rh1^G69D^* (r = 0.12, *P* = 0.13). * *P* < 0.05, ** *P* < 0.005.

Because canonical p53 signaling activates the expression of *rpr*, we expected high correlation in apoptosis levels and eye size between these models (Shlevkov and Morata 2012; Mollereau and Ma 2014). Indeed, there is a significant positive correlation in eye size between DGRP strains expressing *GMR > **p53* and *GMR-rpr* (r = 0.19, *P* = 0.0071) (Figure 3A). In a previous study, we examined the impact of genetic variation on a model of retinitis pigmentosa (RP) and ER stress-induced apoptosis (Chow *et al.* 2016). In that study, we found that the degeneration induced by overexpression of a misfolded protein (*Rh1^G69D^*) in the developing eye imaginal disc is modified by a number of genes involved in apoptosis (Chow *et al.* 2016). This is to be expected, as the primary cause of degeneration in this model is JNK-*hid/grim/**rpr*-mediated cell death (Figure 1) (Kang *et al.* 2012). Consistent with this mechanism of *Rh1^G69D^*-induced degeneration, we found a significant correlation in eye size between the *Rh1^G69D^* and *rpr* models (r = 0.25, *P* = 0.001, Figure 3B). In contrast, we see no correlation between the *Rh1^G69D^* and *p53* models of apoptosis (r = 0.12, *P* = 0.13) (Figure 3C). These results suggest that there is shared genetic architecture between *Rh1^G69D^* and *rpr*-mediated apoptosis and degeneration that is independent from that shared between *p53* and *rpr*.

### rpr-induced degeneration is modified by apoptosis, Wnt signaling, and mitochondrial metabolism

#### Genome-wide association analysis:

To identify the genes driving this phenotypic variability, we performed a genome-wide association analysis to identify genetic polymorphisms that impact the severity of degeneration in the *GMR > **p53* and *GMR-rpr* models of apoptosis. We used mean eye size as a quantitative phenotype to test for association with polymorphisms in the DGRP. With the large number of variants (1,967,719 for *p53* and 1,962,205 for *rpr*) tested for the apoptosis models in ∼200 lines, the analyses were not sufficiently powered for associations to survive multiple testing correction at a false discovery rate of 0.2 (p-value < 1x10^−7^). Previous work from our lab and others, however, have demonstrated the relevance of weak signals obtained from DGRP studies. Specifically, it has been observed that association of candidate genes can be replicated in different lab environments and in different populations (Everman *et al.* 2019; Pitchers *et al.* 2019). Ultimately, our goal is not to treat these associations as definitive, but to use these variants to nominate candidate modifier genes or pathways for subsequent functional validation. Despite the weak signals, this approach has been used to successfully identify *bona fide* modifier genes in previous work (Chow *et al.* 2013, 2015, 2016; Palu and Chow 2018, 2019; Lavoy *et al.* 2018; Ahlers *et al.* 2019). Here again, this genome-wide association analysis yields a number of genes that potentially underlie the variable expressivity of degenerative phenotypes induced by these models of apoptosis. Using an arbitrary p-value cutoff of <1x10^−04^, we identified 128 significantly associated polymorphisms for the *GMR-rpr* model (Table S3). We only considered polymorphisms that fall within an annotated gene or +/− 1 kb of an annotated gene. Polymorphisms located more than 1 kb from an annotated gene were not considered in our analysis. Sixteen polymorphisms lie outside of these parameters and were not considered further. Of the remaining 112 polymorphisms, ten are located in an intergenic region (+/− 1kb), 14 are located in UTRs, 69 are located in introns, and 19 are located in protein-coding sequences. All 19 polymorphisms in coding regions are synonymous variants. These 112 gene-associated polymorphisms lie in 82 candidate genes (Table S3, S4). Sixty-six of the candidate genes have direct human orthologs (Table S4). A more stringent p-value cutoff (<1x10^−5^) yields only 20 polymorphisms, 16 of which lie in 14 candidate genes (12 with human orthologs) (Table S3, S4). Because the more stringent cutoff yielded few candidates, we focused the majority of our analysis on the 82 candidate genes identified at *P* < 1x10^−04^. A less stringent cutoff allows for enrichment analyses. It will also result in more false positives, but, again, we are mainly focused on the genes, and not the variants, as the variants themselves may not be evolutionarily conserved, but the candidate genes themselves may be conserved as modifier genes. Functional analyses of the candidate genes will point to *bona fide* modifiers.

For the *GMR > **p53* model, we identified 24 polymorphisms at a p-value cutoff of <1x10^−04^ (Table S5). Eight of these polymorphisms lie outside of genes and were not considered further. Of the remaining 16 polymorphisms, one is located in a UTR, 15 are located in introns, and eight are intergenic. The 16 gene-associated polymorphisms lie in 13 candidate genes (Table S5, S6). Thirteen of the associated polymorphisms have a p-value of <1x10^−05^. Five of these are intergenic, while the remaining six are in six candidate genes. Interestingly, there is no overlap between the *GMR > **p53* candidate polymorphisms or genes and those identified using the *GMR-rpr* model of apoptosis (Table S3-S6). The only overlap in modifier genes is between *GMR > Rh1^G69D^* and *GMR > **p53* (Table S6) (Chow *et al.* 2016). They share candidate modifier genes *CG31559*, a disulfide oxidoreductase (FlyBase Curators 2004), and *dpr6*, a cell surface immunoglobulin involved in synapse organization (Gaudet *et al.* 2011). It is unclear what the significance of this overlap might be.

We conclude from our initial analysis that the top candidates for our models of degeneration are likely specific to the method by which we induce that degeneration. However, it is also possible that our study is underpowered and we are simply missing overlapping candidate modifiers shared between the two models. Future validation of the candidates will involve specific tests examining the impact of a modifier gene on both apoptotic models.

Even at the lower p-value threshold, there are very few significant associations for *GMR > **p53*, and even fewer in close proximity to a gene. This is likely because the DGRP population, simply due to chance, does not have a great deal of variation affecting the p53 pathway. Because the DGRP was generated from a single population, the entire spectrum of possible variation is simply not represented. It is plausible that screening through an alternate population might yield different and more interesting results for *GMR > **p53* modifiers. With the results reported here, it would be difficult to obtain meaningful results from analyses focused on shared gene ontology, modifier interactions, and shared pathway functions. We therefore elected to focus the remaining analysis on the *GMR-rpr* model.

#### Modifier genes:

Because the *rpr* model is the most direct inducer of apoptosis we have tested, we expected to see apoptotic functions for many of the candidate genes identified in our GWAS. The top hit was the gene *echinus* (*ec*), a ubiquitin specific protease (USP) orthologous to human *USP53* and *USP54* (Table S4). We identified nine intronic SNPs in *ec* through our association analysis. Previous studies show that loss of *ec* in the developing eye results in a mild rough eye phenotype, albeit a much less dramatic one than that seen upon overexpression of *rpr* (Wolff and Ready 1991; Copeland *et al.* 2007). While this previous study reported no genetic interaction between *ec* and *rpr*, this was assessed based on qualitative changes as opposed to quantitative differences in eye size (Copeland *et al.* 2007). Our GWAS data suggests that such a genetic interaction may play an important role in *rpr*-induced degeneration.

*Ec* is one of several apoptotic genes identified in this analysis. In fact, 16/82 (∼20%) of the candidate genes have known functions in apoptosis-related pathways, all of which have conserved human orthologs (Table S4). One of these is *Diap2*, a *Drosophila* paralog of *Diap1* (human orthologs: *BIRC2* and *BIRC3*) (Hay *et al.* 1995). The Diap proteins normally inhibit caspase activation and prevent apoptosis. Expression of the rpr/grim/hid proteins inhibits Diap1 and Diap2, allowing apoptosis to proceed. Increased expression or activity of Diap2 reduces the impact of *rpr* overexpression, thereby reducing apoptosis (Hay *et al.* 1995). Conversely, reduced expression of *Diap2* may not have a strong impact on *rpr*-associated degeneration, as *Diap1* is the major functional paralog in this pathway. The identification of a gene directly involved in the *rpr* pathway demonstrates the efficacy of our GWAS.

Two candidates, *hay* and *Xpd* (*ERCC3* and *ERCC2*) (Table S4), have human orthologs mutated in Xeroderma pigmentosum, an inherited genetic condition where defects in DNA excision repair result in melanomas and eventually death (Kraemer and DiGiovanna 2016). These are subunits of the TFIIH helicase complex that are involved in excision repair after UV damage (Koken *et al.* 1992; Mounkes *et al.* 1992; Reynaud *et al.* 1999). Besides *hay* and *Xpd*, we identified 4 additional genes whose human orthologs are directly involved in cancer: *DIP-iota* (*OPCML*), *Fum4* (*FH*), *CG8405* (*TMEM259*), and *CG15529* (*BLNK*). Mutations in these genes have been associated with ovarian cancer (*OPCML*) (Sellar *et al.* 2003), renal cancer (*FH*) (The Multiple Leiomyoma Consortium 2002; Pollard *et al.* 2005), and various carcinomas (*TMEM259*) (Chen *et al.* 2005). The roles of these genes in cancer are likely due to functions in apoptotic initiation or cell cycle regulation. Other candidates are activated downstream of p53, such as *CG44153* (*ADGRB3*) and *stac* (*BAIAP3*) (Shiratsuchi *et al.* 1997, 1998). This suggests that feedback signaling through p53 can increase *rpr*-induced apoptosis and degeneration.

24/82 candidate genes (∼30%) are involved in neuronal function or implicated in neurological disease. Twenty-three have conserved human orthologs (Table S4). Human orthologs of *Form3* (*INF2*) and *LysRS* (*KARS*) can both be mutated in different forms of the degenerative peripheral neuropathy Charcot-Marie-Tooth disease (McLaughlin *et al.* 2010; Boyer *et al.* 2011), while *Shawl* (*KCNC3*) and *CG7741* (*CWF19L1*) are associated with spinocerebellar ataxia (Waters *et al.* 2006; Burns *et al.* 2014). Mutation in the Rab3-interacting scaffold protein encoded by *Rim* (*RIMS1*) can cause a retinal degenerative disease that is similar to retinitis pigmentosa (Johnson *et al.* 2003), which was the focus of the *Rh1^G69D^* study (Chow *et al.* 2016). Identification of genes with roles in different neuronal and muscular degenerative diseases suggests that these modifiers could be important in a variety of apoptosis-associated diseases.

#### Network analysis:

**Figure 4:**
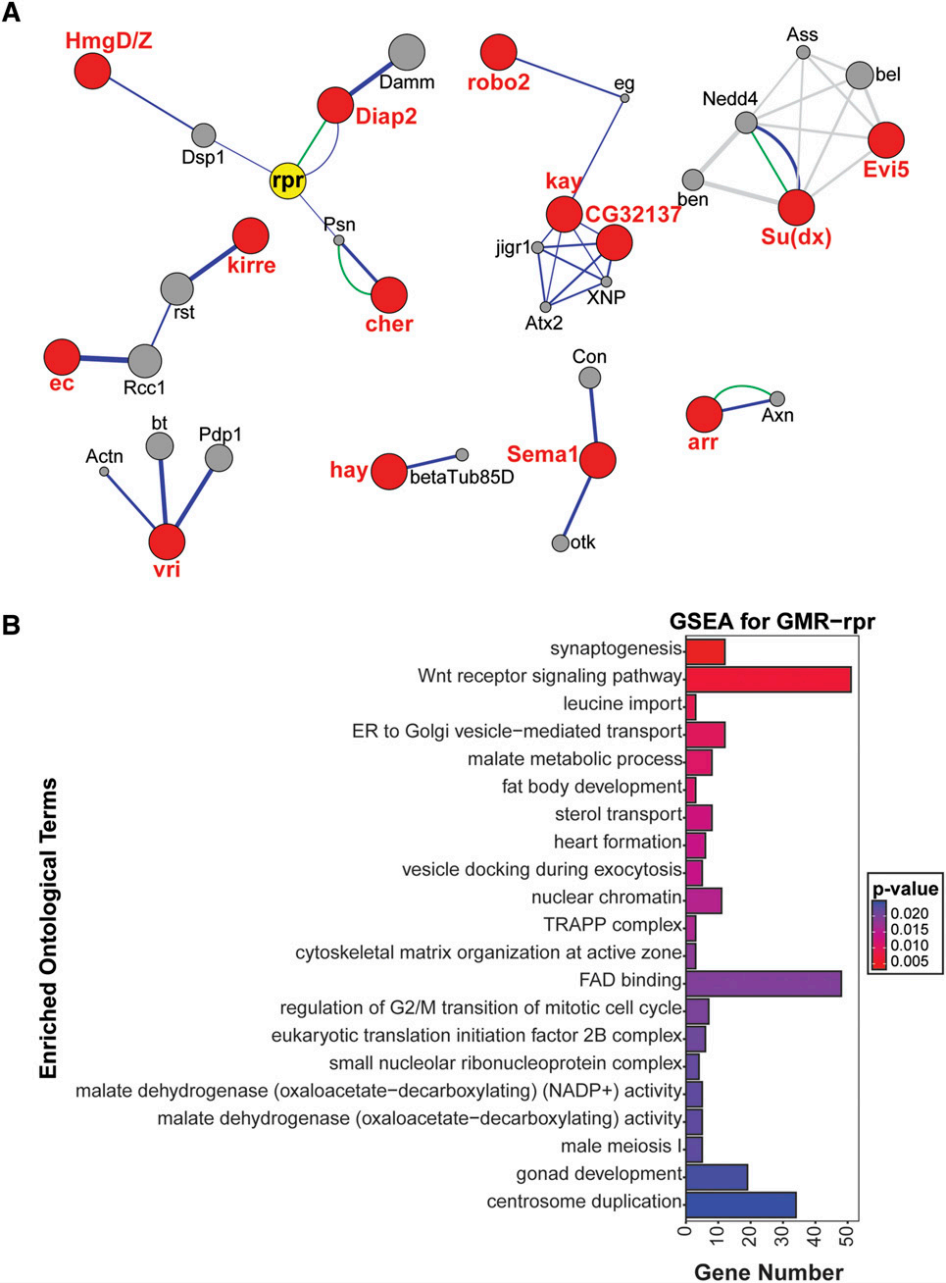
*rpr* modifiers are enriched for neuronal function, Wnt signaling, and metabolic pathways. A. *rpr* modifier network, as plotted by the GeneMANIA plugin in Cytoscape (Shannon *et al.* 2003; Montojo *et al.* 2010). Significant candidate modifiers are indicated in red, with physical interactions shown in green, genetic interactions shown in blue, and predicted interactions shown in gray. Thicker lines indicate stronger evidence for the association. B. Top 20 significant ontological categories as identified by GSEA. Categories are arranged from most significant on top to least significant along the y-axis. P-values are indicated by red-to-blue gradient, with red the lowest p-values and blue the highest P-values. Gene number identified in each category is indicated along the y-axis.

To understand if there are functional relationships between *GMR-rpr* modifiers, we examined interactions among the 82 candidate genes. Genetic, physical, and predicted interactions were compiled and visualized using Cytoscape software (Shannon *et al.* 2003; Montojo *et al.* 2010). Fourteen of the 82 candidate genes were found as nodes in these interaction networks, as was *rpr* itself (Figure 4A). We identified several interesting clusters of candidate genes, including those with functions in apoptosis, development, and protein ubiquitination.

As expected, given the large number of candidates with apoptotic roles, we found an apoptosis cluster of interactions between modifiers with functions associated with cell cycle regulation and cell death (Figure 4A). A number of these genes, including *Diap2* and *cher* (*FLNA*), have either direct or indirect interactions with *rpr* itself. As noted above, *Diap2* interacts both physically and genetically with *rpr* (Figure 1) (Hay *et al.* 1995). *cher* shows indirect genetic interactions with *rpr* through its physical association with the presenillin (*psn*) protein (Guo *et al.* 2000) (Figure 4A). This interaction is conserved in humans, and mutations that alter this interaction are associated with Alzheimer’s Disease (Lu *et al.* 2010).

Among the apoptosis cluster are also regulators of developmental apoptosis in our network, including the *ec* protease (Copeland *et al.* 2007) and the neuronal cell adhesion protein encoded by *kirre* (*KIRREL3*) (Bao *et al.* 2010) (Figure 4A). Indirect genetic interactions were identified between these genes, which are commonly involved in development of the *Drosophila* eye imaginal disc and accompanying regulated apoptosis. The chromatin-binding HmgD/Z (*HMGB2*) proteins are expressed at high levels in the larval CNS, suggesting that they are important for the developmental regulation of neuronal gene expression (Churchill *et al.* 1995; Gaudet *et al.* 2011; Brown *et al.* 2014). They indirectly interact with *rpr* through the closely related *dsp1*, which encodes another paralog of human *HMGB2* (Figure 4A). Dsp1 recruits members of the repressive polycomb complex to chromatin. It is possible that these genetic interactions indicate a role for the HMGB2 proteins in regulating *rpr* expression and, as a result, developmental regulation of cell death and tissue turnover. Our apoptotic model is expressed in a developmental tissue, suggesting that some of the variation in eye size observed across the DGRP could be due to changes in the response of developmental processes to the abnormal activation of apoptosis. Such regulators of developmental apoptosis could be excellent candidates for therapeutic targeting in degenerative diseases.

We also identified a number of predicted interactions in a cluster of modifier genes involved in protein ubiquitination (Figure 4A). Among the top candidate genes are *ec*, *Diap2*, *Su(dx)* (*ITCH*), and *Roc2* (*RNF7*), all of which have important roles in protein degradation through ubiquitination and the proteasome degradation pathway. *Su(dx)*, like *Diap2*, encodes a ubiquitin ligase (Gaudet *et al.* 2011). Our network analysis highlights a predicted interaction between *Su(dx)* and the Rab GTPase-interacting protein *Evi5*, another candidate gene (Laflamme *et al.* 2012) (Figure 4A). This regulator of vesicular fusion is predicted to interact with a number of additional ubiquitin ligases as well (Figure 4A). Degradation of proteins through the proteasome is an important mechanism for maintaining cellular homeostasis under a variety of cellular stresses (Sano and Reed 2013). Altered regulation of E3 ligases, which determine the identity and specificity of proteins for degradation (Ester Morreale and Walden 2016), could tip the balance of cells experiencing apoptotic stress toward or away from cell death.

#### Gene set enrichment analysis:

Thus far, we have focused our analysis on candidate modifiers surviving a specific statistical threshold in our GWAS. While this provides many new avenues for future analysis, it ignores the majority of the association data. We therefore performed gene set enrichment analysis (GSEA), using all GWAS variant data and their associated P-values. The gene nearest to each variant was assigned the variant’s P-value and used as GSEA input, using the method described (Subramanian *et al.* 2005). Given a defined set of genes annotated with a certain GO function, GSEA determines whether the members of that set are randomly distributed throughout the ranked list or if they are found primarily at the top or bottom of that list. GO categories enriched at the top of the list functionally describe the phenotype of the gene set. While traditional GO analysis uses a set of genes based on a P-value cutoff, GSEA examines the entire gene set (Dyer *et al.* 2008). GSEA identified 62 significantly associated gene sets (≥ 3 genes) at a corrected p-value of <0.05 (Table S7). The top gene set was synaptogenesis (GO:0007416, *P* = 3.7 × 10^−3^) and includes *Sema1a* (*SEMA6A*), a conserved semaphorin-binding protein involved in axon guidance (Ayoob *et al.* 2006; Gaudet *et al.* 2011) and one of the top modifier candidates based on individual polymorphism analysis (Figure 4B, Table S7). Other genes in this category include those involved in synapse formation and organization, suggesting that regulating neuronal connectivity and synapse choice could play a role in the decision to apoptose or to survive.

The second most significantly enriched category was Wnt signaling (GO:0016055, *P* = 6.7 × 10^−3^), consisting of 51 enriched genes from our *GMR-rpr* analysis (Figure 4B, Table S7). One of these, *arr*, is also a candidate modifier gene (Table S4,S7). *arr* is a *Drosophila* ortholog of the genes encoding the co-receptors *LRP5/6* in canonical Wnt signaling (Rives *et al.* 2006). The second most significant single candidate gene in the GWA is *swim* (*TINAGL1*/*TINAG*), a secreted cysteine protease capable of binding the wingless (wg) ligand and enhancing its spread and signaling capabilities (Mulligan *et al.* 2012). Also enriched for significant polymorphisms are four frizzled paralogs (Wnt receptors) and six paralogs of the Wnt ligand (Table S7). Other integral components of the canonical Wnt pathway, such as *disheveled*, *axin*, and *CKIα*, are enriched for associated polymorphisms, as are several peripheral and non-canonical regulators of Wnt signaling (Table S7). This striking association is reinforced by previous studies that have linked Wnt signaling with either the promotion or restraint of cell death (Pećina-Slaus 2010). Non-canonical Wnt signaling can activate JNK or calcium release from the ER, both of which can alter the decision to initiate apoptosis (Rasmussen *et al.* 2018). It will be interesting to investigate Wnt signaling collectively as well as with individual candidates to determine how different branches of the pathway impact degenerative diseases.

GSEA also identified a number of genes and pathways involved in mitochondrial homeostasis and metabolism (Figure 4B), including malate metabolic processes (seven genes, GO:0006108, *P* = 0.011). These genes encode for malate dehydrogenase enzymes, six of which are localized to the mitochondrion (Figure 4B, Table S7). Malate dehydrogenase catalyzes the oxidation of malate to oxaloacetate in the last step of the TCA cycle prior to the entrance of acetyl-CoA (Minárik *et al.* 2002). The presence of so many paralogs of this enzyme suggests that mitochondrial metabolism, and in particular the mitochondrial redox state, is a major regulator of apoptosis. Supporting this, one of the top candidates, Fum4 (*FH*), is also an essential enzyme in the TCA cycle (Table S4). The GSEA further supports this finding, as FAD binding is also enriched (48 genes, GO:0050660, *P* = 0.020) (Figure 4B). A primary function for these 48 enriched genes is the maintenance of redox homeostasis, 16 of which localize to the mitochondria. Another of these genes, the apoptosis-inducing factor *AIF*, is activated independently from caspases by mitochondrial stress and is released into the cytoplasm, travels to the nucleus, and initiates the chromatin condensation and DNA fragmentation that immediately precedes cell death (Elmore 2007).

More generally, redox homeostasis in other cellular compartments is also implicated by GSEA (Table S7, Figure 4B). Three paralogs of aldehyde oxidase (*Aox*) and the NAD(P)H oxidoreductase *Duox* (*DUOX1*) are enriched for associated polymorphisms; these oxidase enzymes are essential for maintaining an appropriate balance of reactive oxygen species in the cytoplasm. We identified four paralogs of acyl-coA oxidase (*Acox*), which is involved in the β-oxidation of very long chain fatty acids in the peroxisome, and an additional 4 genes involved in mitochondrial β-oxidation: *wal* (*ETFA*), *Mcad* (*ACADM*), *CG4860* (*ACADS*), and *CG7461* (*ACADVL*).

The involvement of enzymes regulating redox homeostasis, and more specifically redox homeostasis in the mitochondria, is consistent with *rpr*-induced apoptosis. Both caspase-dependent and caspase-independent apoptotic pathways can be activated downstream of mitochondrial stress (Elmore 2007; Rasmussen *et al.* 2018). Increasing the permeability of the mitochondrial membrane is sufficient to ensure activation of the apoptosome through the release of cytochrome-C (Elmore 2007). This, along with expression of the mitochondria-associated IAP inhibitors rpr/grim/hid, activates the caspase cascade (Sandu *et al.* 2010). Damage to the mitochondria that increases permeability, such as through redox stress, is itself sufficient to activate apoptosis in a caspase-independent manner through the release of AIF (Elmore 2007). Importantly, nearly all the mitochondrial genes identified using GSEA analysis are expressed in the imaginal discs (Brown *et al.* 2014). This suggests that mitochondrial function modifies apoptotic progression in a cell autonomous fashion, consistent with the known roles of the mitochondria in cell death.

Other metabolic processes such as sterol transport (GO:0015918, *P* = 0.013), leucine import (GO:0060356, *P* = 8.9 × 10^−3^), and fat body development (GO:0007503, *P* = 0.011) are enriched in the GSEA (Table S7, Figure 4B). Disruption of metabolic processes has long been known to induce oxidative and ER stress, both of which are capable of activating apoptosis through JNK/*grm-rpr-hid* signaling cascades or directly through mitochondrial stress (Kanda and Miura 2004). It will be interesting to explore how these metabolic processes alter apoptosis not only in this model of retinal degeneration, but in physiologically relevant cell types and tissues, such as the midgut, fat body, and insulin-producing cells.

The enrichment of multiple metabolic categories suggests that the impact of cellular and mitochondrial metabolism on redox homeostasis could play a major role in *rpr*-induced degeneration. We hypothesize that these regulators of mitochondrial redox state and metabolism are directly and indirectly influencing the activation of mitochondrial proteins involved in the final decision to undergo apoptosis. Our GSEA emphasizes the importance of exploring not just individually associated genes but also their functional pathways and partners when identifying genetic modifiers of disease.

### Functional analysis of candidate modifiers of apoptosis

**Figure 5:**
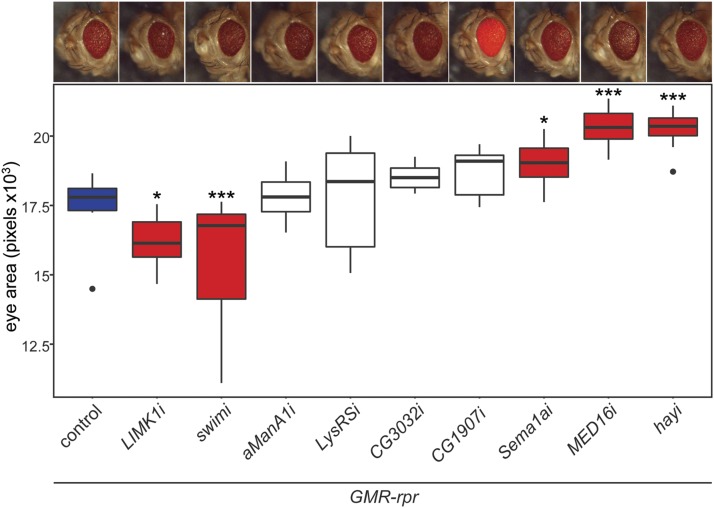
Knockdown of candidate *rpr* modifiers significantly alters apoptosis-induced degeneration. RNAi against candidate modifiers was expressed under the control of *GMR-GAL4* in the *GMR-rpr* model. The genetically matched attP2 line was crossed into the *GMR-rpr* line as a control (blue). Eye size in pixels was quantified for N = 11-15 flies per strain and plotted with the 25^th^-75^th^ percentile of measurements in the central box. Measurements lying outside of 1.5 × interquartile range are indicated as points. Representative images of each line are found above the data for that line. Knockdown of *LIMK1* or *swim* significantly reduces eye size in the *GMR-rpr* model of degeneration compared to controls. Loss of *Sema1a*, *MED16*, or *hay* results in a significant increase in eye size compared to controls. Loss of *CG1907* does not significantly alter eye size, but changes in pigmentation are similar in the presence or absence of *GMR-rpr* (Fig S4C). Loss of *αManA1*, *LysRS*, or *CG3032* do not produce a significant effect. RNAi lines with significant changes in eye size are indicated in red, while those that are not significantly changed are indicated in white. * *P* < 0.05, *** *P* < 0.0005.

To confirm the roles of our candidate genes in regulating apoptosis, we elected to test the impact of loss of modifier expression for the top nine *rpr* candidate genes and the top seven *p53* candidate genes for which we were able to obtain transgenic RNAi lines. We crossed the RNAi strains targeting each of these modifiers into the *GMR-rpr* or *GMR > **p53* line, and then measured the eye area in offspring carrying both the RNAi construct and the apoptosis model (Figure 5, Figure S3). Eye area was quantified and compared to a genetically matched control expressing only the apoptosis model (Figure S4A). We also crossed the RNAi lines to a strain expressing only GMR-GAL4 as a control for independent modifier effect. If loss of the candidate alone has a substantial impact on eye size or phenotype, it would suggest that this candidate is NOT specific to the model in question.

For modifiers of *p53*-induced apoptosis, we found that knockdown of *Ir40A* (15952 ± 716 pixels, N = 15) expression resulted in enhancement of the apoptosis phenotype, showing a significant decrease in eye size compared to controls expressing only *GMR > **p53* (17459 ± 492 pixels, N = 9) (Figure S3A). Knockdown of *shab* (*KCNB1*) (16598 ± 1143 pixels, N = 10), *CycE* (*CCNE1*) (17462 ± 581 pixels, N = 15), or *bru1* (*CELG2*) (16598 ± 1143 pixels, N = 10), resulted in a partial rescue, with a significant increase in eye size compared to controls expressing *GMR > **p53* (18022 ± 884 pixels, N = 15) (Figure S3B). However, knockdown of either *CycE* or *bru1* in the *GMR-GAL4* (no apoptosis control) background was also sufficient to increase eye size (Figure S3C), suggesting that these modifiers might be working independently from *p53* overexpression. No significant change in eye size was observed upon knockdown of *cyt-c-P* (*CYCS*) (16836 ± 1052 pixels, N = 15), *CG31559* (*GRXCR1*) (17102 ± 934 pixels, N = 15), or *sif* (*TIAM1*) (17218 ± 939 pixels, N = 15) as compared to controls expressing only *GMR > **p53* (17459 ± 492 pixels, N = 9) (Figure S3A). While some of these genes may still be interesting modifiers that would benefit from follow-up studies, our results suggest that the modifiers identified in the *GMR > **p53* screen are either of weak individual effect or, potentially, false positives. This also demonstrates the importance of validation studies such as these when analyzing GWA candidates.

We next focused our analysis on the *rpr* modifiers. Knockdown of either *LIMK1* (*LIMK1*) (16183 ± 875 pixels, N = 15) or *swim* expression (15518 ± 2418 pixels, N = 14) resulted in enhancement of the apoptosis phenotype, showing a significant decrease in eye size compared to controls expressing only *GMR-rpr* (17534 ± 1098 pixels, N = 11) (Figure 5). Knockdown of *sema1a* (18990 ± 746 pixels, N = 15), *MED16* (*MED16*) (20323 ± 622 pixels, N = 15), or *hay* (20240 ± 617 pixels, N = 14) resulted in a partial rescue, with a significant increase in eye size compared to controls expressing *GMR-rpr* (Figure 5). No significant change in eye size was observed upon knockdown of *CG3032* (*GZF1*) (18525 ± 449 pixels, N = 12), *LysRS* (*KARS*) (17879 ± 1834 pixels, N = 12), *αMan-1A* (*MAN1A2*) (17842 ± 763 pixels, N = 15), or *CG1907* (*SLC25A11*) (18755 ± 787 pixels, N = 13) (Figure 5). In the absence of *rpr* overexpression, RNAi revealed no significant change in phenotype from controls (Figure S4B,C). These results suggest that many of the top GWA candidate modifiers are capable of modifying the apoptotic phenotypes associated with the *GMR-rpr* model of degeneration.

It is of course theoretically possible that we may have observed a positive result for any five of nine randomly selected genes within the genome due to the fact that the genetic background is sensitized by the over-expression of rpr. Our results with the similar *GMR > **p53* model suggest that this is not likely to be the case. We examined seven candidate *p53* modifiers of similar significance as the *GMR-rpr* candidates and observed only two that specifically affect eye size when apoptosis is activated. This suggests that while, as previously postulated, the *GMR > **p53* candidates could be false positives, the *GMR-rpr* candidates are more likely to be true positives. Other groups using the DGRP have found this to be true as well (Vonesch *et al.* 2016).

In the future we will also examine the impact of overexpression of candidate genes on the *GMR-rpr* model of apoptosis, as some candidate genes may exert a stronger influence under conditions of gain of function, rather than loss of function.

## Conclusions

The primary goal of this study was to identify candidate genes and pathways that modify apoptosis and degenerative processes. Apoptosis is a primary cause of disease in a multitude of degenerative disorders (Mattson 2000). It is also a commonly targeted pathway for cancer therapies (Ouyang *et al.* 2012). These and other diseases are subject to a large degree of phenotypic heterogeneity due to inter-individual differences in genetic background among patients (Queitsch *et al.* 2012; Chow 2016). Our results point to stress-associated signals as important modifiers of apoptosis-induced degeneration. Overexpression of the apoptotic gene *rpr* can be induced transcriptionally by either p53 or by stress-associated transcription factors such as Jun (Shlevkov and Morata 2012). Many of the candidate modifiers of the *GMR-rpr* model feed into the stress response through mitochondrial metabolism, Wnt signaling, and protein degradation. Their identification as modifier pathways indicates that *rpr* activity is being modulated by feedback signals through these pathways and suggests that apoptotic diseases might be targeted through these stress-related pathways. Understanding how genetic diversity in the population impacts apoptosis could therefore lead to identification of prognostic predictors in the diagnosis of disease and of new therapeutic targets. The modifiers identified here inform our understanding of cell death regulation and could serve as therapeutic targets in a variety of apoptosis-related disorders.

## References

[bib1] Ahlers L. R. H., Trammell C. E., Carrell G. F., Mackinnon S., Torrevillas B. K., 2019 Insulin potentiates JAK/STAT signaling to broadly inhibit flavivirus replication in insect vectors. bioRxiv 701714 10.1101/701714 PMC6871768 31722209

[bib2] Ayoob J. C., Terman J. R., and Kolodkin A. L., 2006 Drosophila Plexin B is a Sema-2a receptor required for axon guidance. Development 133: 2125–2135. 10.1242/dev.02380 16672342

[bib3] Bao S., Fischbach K.-F., Corbin V., and Cagan R. L., 2010 Preferential adhesion maintains separation of ommatidia in the Drosophila eye. Dev. Biol. 344: 948–956. 10.1016/j.ydbio.2010.06.013 20599904 PMC2921583

[bib4] Boyer O., Nevo F., Plaisier E., Funalot B., Gribouval O., 2011 *INF2* Mutations in Charcot–Marie–Tooth Disease with Glomerulopathy. N. Engl. J. Med. 365: 2377–2388. 10.1056/NEJMoa1109122 22187985

[bib5] Brown J. B., Boley N., Eisman R., May G. E., Stoiber M. H., 2014 Diversity and dynamics of the Drosophila transcriptome. Nature 512: 393–399. 10.1038/nature12962 24670639 PMC4152413

[bib6] Burns R., Majczenko K., Xu J., Peng W., Yapici Z., 2014 Homozygous splice mutation in CWF19L1 in a Turkish family with recessive ataxia syndrome. Neurology 83: 2175–2182. 10.1212/WNL.0000000000001053 25361784 PMC4276403

[bib7] Chen Y.-C., Davidson B., Cheng C.-C., Maitra A., Giuntoli R. L., 2005 Identification and characterization of membralin, a novel tumor-associated gene, in ovarian carcinoma. Biochim. Biophys. Acta - Gene Struct. Expr. 1730: 96–102. 10.1016/J.BBAEXP.2005.06.008 16084606

[bib8] Chow C. Y., Wolfner M. F., and Clark A. G., 2013 Using natural variation in Drosophila to discover previously unknown endoplasmic reticulum stress genes. Proc. Natl. Acad. Sci. USA 110: 9013–9018. 10.1073/pnas.1307125110 23667151 PMC3670321

[bib9] Chow C. Y., Avila F. W., Clark A. G., and Wolfner M. F., 2015 Induction of excessive Endoplasmic reticulum stress in the Drosophila male accessory gland results in infertility. PLoS One 10: e0119386 10.1371/journal.pone.0119386 25742606 PMC4350844

[bib10] Chow C. Y., 2016 Bringing genetic background into focus. Nat. Rev. Genet. 17: 63–64. 10.1038/nrg.2015.9 26659016

[bib11] Chow C. Y., Kelsey K. J. P., Wolfner M. F., and Clark A. G., 2016 Candidate genetic modifiers of retinitis pigmentosa identified by exploiting natural variation in Drosophila. Hum. Mol. Genet. 25: 651–659. 10.1093/hmg/ddv502 26662796 PMC4743685

[bib12] Chow C. Y., and Reiter L. T., 2017 Etiology of Human Genetic Disease on the Fly. Trends Genet. 33: 391–398. 10.1016/j.tig.2017.03.007 28420493

[bib13] Churchill M. E., Jones D. N., Glaser T., Hefner H., Searles M. A., 1995 HMG-D is an architecture-specific protein that preferentially binds to DNA containing the dinucleotide TG. EMBO J. 14: 1264–1275. 10.1002/j.1460-2075.1995.tb07110.x 7720717 PMC398206

[bib14] Copeland J. M., Bosdet I., Freeman J. D., Guo M., Gorski S. M., 2007 echinus, required for interommatidial cell sorting and cell death in the Drosophila pupal retina, encodes a protein with homology to ubiquitin-specific proteases. BMC Dev. Biol. 7: 82 10.1186/1471-213X-7-82 17612403 PMC1950886

[bib15] Dyer M. D., Murali T. M., and Sobral B. W., 2008 The Landscape of Human Proteins Interacting with Viruses and Other Pathogens. PLoS Pathog. 4: e32 10.1371/journal.ppat.0040032 18282095 PMC2242834

[bib16] Elmore S., 2007 Apoptosis: a review of programmed cell death. Toxicol. Pathol. 35: 495–516. 10.1080/01926230701320337 17562483 PMC2117903

[bib17] Ester Morreale F., and Walden H., 2016 Types of Ubiquitin Ligases. 10.1016/j.cell.2016.03.003 27015313

[bib18] Everman E. R., McNeil C. L., Hackett J. L., Bain C. L., and Macdonald S. J., 2019 Dissection of Complex, Fitness-Related Traits in Multiple Drosophila Mapping Populations Offers Insight into the Genetic Control of Stress Resistance. Genetics 211: 1449–1467. 10.1534/genetics.119.301930 30760490 PMC6456312

[bib19] FlyBase Curators, Swiss-Prot Project Members, and InterPro Project Members, 2004 Gene Ontology annotation in FlyBase through association of InterPro records with GO terms.

[bib20] Gallati S., 2014 Disease-modifying genes and monogenic disorders: experience in cystic fibrosis. Appl. Clin. Genet. 7: 133–146. 10.2147/TACG.S18675 25053892 PMC4104546

[bib21] Gaudet P., Livstone M., and Thomas P., 2011 Phylogenetic-based propagation of functional annotations within the Gene Ontology consortium. Brief. Bioinform. 12: 449–462. 10.1093/bib/bbr042 21873635 PMC3178059

[bib22] Guo Y., Zhang S. X., Sokol N., Cooley L., and Boulianne G. L., 2000 Physical and genetic interaction of filamin with presenilin in Drosophila. J. Cell Sci. 113: 3499–3508. 10984440 10.1242/jcs.113.19.3499

[bib23] Hay B. A., Wassarman D. A., and Rubin G. M., 1995 Drosophila homologs of baculovirus inhibitor of apoptosis proteins function to block cell death. Cell 83: 1253–1262. 10.1016/0092-8674(95)90150-7 8548811

[bib24] He B. Z., Ludwig M. Z., Dickerson D. A., Barse L., Arun B., 2014 Effect of Genetic Variation in a *Drosophila* Model of Diabetes-Associated Misfolded Human Proinsulin. Genetics 196: 557–567. 10.1534/genetics.113.157800 24281155 PMC3914626

[bib25] Huang W., Massouras A., Inoue Y., Peiffer J., Ràmia M., 2014 Natural variation in genome architecture among 205 Drosophila melanogaster Genetic Reference Panel lines. Genome Res. 24: 1193–1208. 10.1101/gr.171546.113 24714809 PMC4079974

[bib26] Jin S., Martinek S., Joo W. S., Wortman J. R., Mirkovic N., 2000 Identification and characterization of a p53 homologue in Drosophila melanogaster. PNAS 97: 7301–7306. 10.1073/pnas.97.13.7301 10860994 PMC16540

[bib27] Johnson S., Halford S., Morris A. G., Patel R. J., Wilkie S. E., 2003 Genomic organisation and alternative splicing of human RIM1, a gene implicated in autosomal dominant cone-rod dystrophy (CORD7). Genomics 81: 304–314. 10.1016/S0888-7543(03)00010-7 12659814

[bib28] Kanda H., and Miura M., 2004 Regulatory Roles of JNK in Programmed Cell Death. J. Biochem. 136: 1–6. 10.1093/jb/mvh098 15269233

[bib29] Kang M. J., Chung J., and Ryoo H. D., 2012 CDK5 and MEKK1 mediate pro-apoptotic signalling following endoplasmic reticulum stress in an autosomal dominant retinitis pigmentosa model. Nat. Cell Biol. 14: 409–415. 10.1038/ncb2447 22388889 PMC3319494

[bib30] Koken M. H. M., Vreeken C., Bol S. A. M., Cheng N. C., Jaspers-Dekker I., 1992 Cloning and characterization of the *Drosophila* homolog of the xeroderma pigmentosum complementation-group B correcting gene, *ERCC3*. Nucleic Acids Res. 20: 5541–5548. 10.1093/nar/20.21.5541 1454518 PMC334384

[bib31] Kraemer K. H., and DiGiovanna J. J., 2016 Xeroderma Pigmentosum

[bib32] Kurtishi A., Rosen B., Patil K. S., Alves G. W., and Møller S. G., 2018 Cellular Proteostasis in Neurodegeneration. Mol. Neurobiol. 10.1007/s12035-018-1334-z 30182337

[bib33] Laflamme C., Assaker G., Ramel D., Dorn J. F., She D., 2012 Evi5 promotes collective cell migration through its Rab-GAP activity. J. Cell Biol. 198: 57–67. 10.1083/jcb.201112114 22778279 PMC3392932

[bib34] Lavoy S., Chittoor-Vinod V. G., Chow C. Y., and Martin I., 2018 Genetic Modifiers of Neurodegeneration in a Drosophila Model of Parkinson’s Disease. Genetics 209: 1345–1356. 10.1534/genetics.118.301119 29907646 PMC6063243

[bib35] Lu Q., Ding K., Frosch M. P., Jones S., Wolfe M., 2010 Alzheimer’s disease-linked presenilin mutation (PS1M146L) induces filamin expression and γ-secretase independent redistribution. J. Alzheimers Dis. 22: 235–245. 10.3233/JAD-2010-100585 20847418 PMC3813954

[bib36] Mackay T. F. C., Richards S., Stone E. A., Barbadilla A., Ayroles J. F., 2012 The Drosophila melanogaster Genetic Reference Panel. Nature 482: 173–178. 10.1038/nature10811 22318601 PMC3683990

[bib37] Mattson M. P., 2000 Apoptosis in neurodegenerative disorders. Nat. Rev. Mol. Cell Biol. 1: 120–130. 10.1038/35040009 11253364

[bib38] McLaughlin H. M., Sakaguchi R., Liu C., Igarashi T., Pehlivan D., 2010 Compound Heterozygosity for Loss-of-Function Lysyl-tRNA Synthetase Mutations in a Patient with Peripheral Neuropathy. Am. J. Hum. Genet. 87: 560–566. 10.1016/j.ajhg.2010.09.008 20920668 PMC2948804

[bib39] Minárik P., Tomášková N., Kollárová M., and Antalík M., 2002 *Malate Dehydrogenases-Structure and Function*. 12537350

[bib40] Mollereau B., and Ma D., 2014 The p53 control of apoptosis and proliferation: lessons from Drosophila. Apoptosis 19: 1421–1429. 10.1007/s10495-014-1035-7 25217223 PMC4167030

[bib41] Montojo J., Zuberi K., Rodriguez H., Kazi F., Wright G., 2010 GeneMANIA Cytoscape plugin: fast gene function predictions on the desktop. Bioinformatics 26: 2927–2928. 10.1093/bioinformatics/btq562 20926419 PMC2971582

[bib42] Mounkes L. C., Jones R. S., Liang B.-C., Gelbart W., and Fuller M. T., 1992 A Drosophila model for xeroderma pigmentosum and Cockayne’s syndrome: haywire encodes the fly homolog of ERCC3, a human excision repair gene. Cell 71: 925–937. 10.1016/0092-8674(92)90389-T 1458540

[bib43] Mulligan K. A., Fuerer C., Ching W., Fish M., Willert K., 2012 Secreted Wingless-interacting molecule (Swim) promotes long-range signaling by maintaining Wingless solubility. Proc. Natl. Acad. Sci. USA 109: 370–377. 10.1073/pnas.1119197109 22203956 PMC3258625

[bib44] Nadeau J. H., 2001 Modifier genes in mice and humans. Nat. Rev. Genet. 2: 165–174. 10.1038/35056009 11256068

[bib45] Ouyang L., Shi Z., Zhao S., Wang F.-T., Zhou T.-T., 2012 Programmed cell death pathways in cancer: a review of apoptosis, autophagy and programmed necrosis. Cell Prolif. 45: 487–498. 10.1111/j.1365-2184.2012.00845.x 23030059 PMC6496669

[bib46] Palu R. A. S., and Chow C. Y., 2018 Baldspot/ELOVL6 is a conserved modifier of disease and the ER stress response. PLoS Genet. 14: e1007557 10.1371/journal.pgen.1007557 30081392 PMC6078684

[bib47] Palu R. A. S., and Chow C. Y., 2019 The Drosophila metallopeptidase superdeath decouples apoptosis from the activation of the ER stress response. bioRxiv 620492 10.1101/620492 PMC7153941 32047096

[bib48] Pećina-Slaus N., 2010 Wnt signal transduction pathway and apoptosis: a review. Cancer Cell Int. 10: 22 10.1186/1475-2867-10-22 20591184 PMC2908610

[bib49] Pitchers W., Nye J., Márquez E. J., Kowalski A., Dworkin I., 2019 A multivariate genome-wide association study of wing shape in drosophila melanogaster. Genetics 211: 1429–1447. 10.1534/genetics.118.301342 30792267 PMC6456314

[bib50] Pollard P. J., Brière J. J., Alam N. A., Barwell J., Barclay E., 2005 Accumulation of Krebs cycle intermediates and over-expression of HIF1α in tumours which result from germline FH and SDH mutations. Hum. Mol. Genet. 14: 2231–2239. 10.1093/hmg/ddi227 15987702

[bib51] Queitsch C., Carlson K. D., and Girirajan S., 2012 Lessons from model organisms: phenotypic robustness and missing heritability in complex disease. PLoS Genet. 8: e1003041 10.1371/journal.pgen.1003041 23166511 PMC3499356

[bib52] Rasmussen M., Ortolano N., Romero-Morales A., Gama V., Rasmussen M. L., 2018 Wnt Signaling and Its Impact on Mitochondrial and Cell Cycle Dynamics in Pluripotent Stem Cells. Genes (Basel) 9: 109 10.3390/genes9020109 PMC5852605 29463061

[bib53] Reynaud E., Lomelí H., Vázquez M., and Zurita M., 1999 The Drosophila melanogaster Homologue of the Xeroderma Pigmentosum D Gene Product Is Located in Euchromatic Regions and Has a Dynamic Response to UV Light-induced Lesions in Polytene Chromosomes. Mol. Biol. Cell 10: 1191–1203. 10.1091/mbc.10.4.1191 10198066 PMC25250

[bib54] Rives A. F., Rochlin K. M., Wehrli M., Schwartz S. L., and DiNardo S., 2006 Endocytic trafficking of Wingless and its receptors, Arrow and DFrizzled-2, in the Drosophila wing. Dev. Biol. 293: 268–283. 10.1016/j.ydbio.2006.02.006 16530179 PMC7897421

[bib55] Sandu C., Ryoo H. D., and Steller H., 2010 Drosophila IAP antagonists form multimeric complexes to promote cell death. J. Cell Biol. 190: 1039–1052. 10.1083/jcb.201004086 20837774 PMC3101595

[bib56] Sano R., and Reed J. C., 2013 ER stress-induced cell death mechanisms. Biochim. Biophys. Acta 1833: 3460–3470. 10.1016/j.bbamcr.2013.06.028 23850759 PMC3834229

[bib57] Scriver C. R., and Waters P. J., 1999 Monogenic traits are not simple: lessons from phenylketonuria. Trends Genet. 15: 267–272. 10.1016/S0168-9525(99)01761-8 10390625

[bib58] Sellar G. C., Watt K. P., Rabiasz G. J., Stronach E. A., Li L., 2003 OPCML at 11q25 is epigenetically inactivated and has tumor-suppressor function in epithelial ovarian cancer. Nat. Genet. 34: 337–343. 10.1038/ng1183 12819783

[bib59] Shannon P., Markiel A., Ozier O., Baliga N. S., Wang J. T., 2003 Cytoscape: a software environment for integrated models of biomolecular interaction networks. Genome Res. 13: 2498–2504. 10.1101/gr.1239303 14597658 PMC403769

[bib60] Shiratsuchi T., Nishimori H., Ichise H., Nakamura Y., and Tokino T., 1997 Cloning and characterization of BAI2 and BAI3, novel genes homologous to brain-specific angiogenesis inhibitor 1 (BAM). Cytogenet. Genome Res. 79: 103–108. 10.1159/000134693 9533023

[bib61] Shiratsuchi T., Oda K., Nishimori H., Suzuki M., Takahashi E., 1998 Cloning and Characterization of BAP3 (BAI-Associated Protein 3), a C2 Domain-Containing Protein That Interacts with BAI1. Biochem. Biophys. Res. Commun. 251: 158–165. 10.1006/bbrc.1998.9408 9790924

[bib62] Shklover J., Levy-Adam F., and Kurant E., 2015 The role of Drosophila TNF Eiger in developmental and damage-induced neuronal apoptosis. FEBS Lett. 589: 871–879. 10.1016/j.febslet.2015.02.032 25754009

[bib63] Shlevkov E., and Morata G., 2012 A dp53/JNK-dependant feedback amplification loop is essential for the apoptotic response to stress in Drosophila. Cell Death Differ. 19: 451–460. 10.1038/cdd.2011.113 21886179 PMC3278728

[bib64] Subramanian A., Tamayo P., Mootha V. K., Mukherjee S., Ebert B. L., 2005 Gene set enrichment analysis: a knowledge-based approach for interpreting genome-wide expression profiles. Proc. Natl. Acad. Sci. USA 102: 15545–15550. 10.1073/pnas.0506580102 16199517 PMC1239896

[bib65] The Multiple Leiomyoma Consortium, 2002 Germline mutations in FH predispose to dominantly inherited uterine fibroids, skin leiomyomata and papillary renal cell cancer. Nat. Genet. 30: 406–410. 10.1038/ng849 11865300

[bib66] Vonesch S. C., Lamparter D., Mackay T. F. C., Bergmann S., and Hafen E., 2016 Genome-Wide Analysis Reveals Novel Regulators of Growth in Drosophila melanogaster. PLoS Genet. 12: e1005616 10.1371/journal.pgen.1005616 26751788 PMC4709145

[bib67] Waters M. F., Minassian N. A., Stevanin G., Figueroa K. P., Bannister J. P. A., 2006 Mutations in voltage-gated potassium channel KCNC3 cause degenerative and developmental central nervous system phenotypes. Nat. Genet. 38: 447–451. 10.1038/ng1758 16501573

[bib68] Wolff T., and Ready D. F., 1991 Cell death in normal and rough eye mutants of Drosophila. Development 113: 825–839 https://doi.org/8223268. 1821853 10.1242/dev.113.3.825

[bib69] Zhou X., and Stephens M., 2012 Genome-wide efficient mixed-model analysis for association studies. Nat. Genet. 44: 821–824. 10.1038/ng.2310 22706312 PMC3386377

